# The Relationship Between Fractures and DXA Measures of BMD in the Distal Femur of Children and Adolescents With Cerebral Palsy or Muscular Dystrophy

**DOI:** 10.1359/jbmr.091007

**Published:** 2009-10-12

**Authors:** Richard C Henderson, Lisa M Berglund, Ryan May, Babette S Zemel, Richard I Grossberg, Julie Johnson, Horacio Plotkin, Richard D Stevenson, Elizabeth Szalay, Brenda Wong, Heidi H Kecskemethy, H Theodore Harcke

**Affiliations:** 1 Departments of Orthopaedics and Pediatrics, University of North CarolinaChapel Hill, NC, USA; 2 Department of Orthopaedics, University of North CarolinaChapel Hill, NC, USA; 3 Department of Biostatistics, University of North CarolinaChapel Hill, NC, USA; 4 Department of Pediatrics, University of Pennsylvania School of Medicine, Children's Hospital of PhiladelphiaPhiladelphia, PA, USA; 5 Hattie Larlham Center for Children with DisabilitiesMantua, OH, USA; 6 Children's Care Hospital and SchoolSioux Falls, SD, USA; 7 Departments of Pediatrics and Orthopedic Surgery, University of Nebraska Medical CenterOmaha, NE, USA; 8 Department of Pediatrics, University of VirginiaCharlottesville, VA, USA; 9 Department of Orthopaedics, University of New MexicoAlbuquerque, NM, USA; 10 Departments of Pediatrics and Neurology, Cincinnati Children's Hospital Medical CenterCincinnati, OH, USA; 11 Nemours Biomedical Research and Department of Medical Imaging, A. I. duPont Hospital for ChildrenWilmington, DE, USA; 12 Department of Medical Imaging, A. I. duPont Hospital for ChildrenWilmington, DE, USA

**Keywords:** Fractures, Osteopenia, Children, Bone Density, Disabilities

## Abstract

Children with limited or no ability to ambulate frequently sustain fragility fractures. Joint contractures, scoliosis, hip dysplasia, and metallic implants often prevent reliable measures of bone mineral density (BMD) in the proximal femur and lumbar spine, where BMD is commonly measured. Further, the relevance of lumbar spine BMD to fracture risk in this population is questionable. In an effort to obtain bone density measures that are both technically feasible and clinically relevant, a technique was developed involving dual-energy X-ray absorptiometry (DXA) measures of the distal femur projected in the lateral plane. The purpose of this study is to test the hypothesis that these new measures of BMD correlate with fractures in children with limited or no ability to ambulate. The relationship between distal femur BMD *Z*-scores and fracture history was assessed in a cross-sectional study of 619 children aged 6 to 18 years with muscular dystrophy or moderate to severe cerebral palsy compiled from eight centers. There was a strong correlation between fracture history and BMD *Z*-scores in the distal femur; 35% to 42% of those with BMD *Z*-scores less than −5 had fractured compared with 13% to 15% of those with BMD *Z*-scores greater than −1. Risk ratios were 1.06 to 1.15 (95% confidence interval 1.04–1.22), meaning a 6% to 15% increased risk of fracture with each 1.0 decrease in BMD *Z*-score. In clinical practice, DXA measure of BMD in the distal femur is the technique of choice for the assessment of children with impaired mobility. © 2010 American Society for Bone and Mineral Research

## Introduction

It is well established that dual-energy X-ray absorptiometry (DXA) measures of bone mineral density (BMD) in elderly adults correlate with fracture risk, but only relatively recently have studies examined the relationship between DXA measures of BMD and fractures in children. Single-observation cross-sectional studies of apparently healthy children have found a correlation between DXA measures in the lumbar spine and previous fractures.(1,2) Various DXA measures also have been found to prospectively predict future fracture risk in healthy children.(3,4)

Children with physical disabilities that limit ambulation typically have low BMD, and many will sustain fractures.(5–7) Compared with healthy children, the fractures in children with disabilities are quite different. The most common site for fractures in healthy children is the forearm, with over 80% of the fractures occurring in the upper limb and fewer than 2% in the femur.(8) In contrast, over 70% of fractures occur in the lower limb of nonambulatory children with disabilities such as cerebral palsy (CP) or Duchenne muscular dystrophy (MD), and up to one-half of all fractures are in the femur.(7,9,10) In healthy children, the most common mechanism of injury is a fall, often from greater than standing height or while running. In children with disabilities, the fractures can occur with minimal trauma that may not even be recognized. Fractures in children with disabilities also differ significantly from those in osteoporotic elderly adults, in whom spinal compression fractures are quite prevalent yet are extremely rare in nonambulatory children.

Given these differences between healthy children, children with disabilities that limit ambulation, and osteoporotic elderly adults, one should not assume that measures of BMD necessarily will relate to fracture in the same way in all three groups. In fact, DXA measures of BMD in the lumbar spine were not found to predict subsequent fracture risk in a small series of children with quadriplegic CP.(11) Further, joint contractures, scoliosis, hip dysplasia, and metallic implants frequently prevent reliable measures of BMD in the proximal femur and lumbar spine, where BMD is most commonly measured. In an effort to obtain bone measures that are both technically feasible and clinically relevant, a new technique was developed involving DXA measures of the distal femur projected in the lateral plane.(12,13)

Other studies have assessed the multiple factors that may contribute to low BMD in children with physical disabilities.(5,7,14–17) In contrast to these studies, the purpose of this study is to test the hypothesis that the new measure of BMD in the distal femur correlates with fractures in children with limited or no ability to ambulate.

## Subjects and Methods

Eight centers using the distal femur scan technique were asked to submit data on all patients or research subjects who had undergone a distal femur DXA scan at their center and met the following criteria: (1) aged 6.0 to 18.0 years at the time of the DXA scan, matching the age range of the normal reference data,(18) and (2) either CP of sufficient severity to significantly impair ambulation or Duchenne MD. Children with conditions in addition to CP or MD that may affect bone metabolism were excluded, as were children who had received bisphosphonate treatment for osteopenia prior to the DXA scan. Routine clinical care of children with these conditions typically includes physical therapy involving weight-bearing activities, and care of all children should include ensuring adequate calcium and vitamin D. Prior use of these interventions was not considered an exclusion criterion. Children with CP frequently have seizure disorder for which they are given anticonvulsants; this was not considered an exclusion criterion. Similarly, children with MD frequently are treated with glucocorticoids, and this too was not considered an exclusion criterion. Selection criteria generally were broad so as to include the spectrum of such children one may encounter in clinical practice and to provide a range of BMD values that would be adequate in assessing the relationship between BMD and fracture risk.

A total of 507 subjects with CP and 112 with MD met these criteria (Table 1). A DXA scan of the lumbar spine was obtained at the same time as the distal femur scan on 229 of these subjects, 179 with CP and 50 with MD. Lumbar spine scans were obtained on only a limited number of subjects for multiple reasons, including distorted anatomy (scoliosis), metallic fixation, and the opinion at some centers that lumbar spine DXA scans are of limited relevance to fracture risk in this population. The study group had a mean age of 11.8 ± 3.4 years (± SD) and was 78% white and 15% black, and 46% of the CP subjects were female (all MD subjects were male).

**Table 1:** Individual Sites' Contributions to the Study Group

		Subjects			
				
Site		CP	DMD	Usual indication for scans	BMD *Z*-score^a^
University of North Carolina	Total	82		Research	−3.8 ± 2.4
	# Yes fx	24			
	% fx	29%			
A. I. duPont Hospital for Children	Total	151	51	Clinical	−4.7 ± 3.4
	# Yes fx	70	14		
	% fx	46%	27%		
University of Nebraska	Total	30		Clinical	−4.8 ± 2.1
	# Yes fx	13			
	% fx	43%			
University of New Mexico	Total	31		Clinical	−4.5 ± 3.2
	# Yes fx	5			
	% fx	16%			
Cincinnati Children's Hospital	Total		61	Clinical	−3.8 ± 2.8
	# Yes fx		5		
	% fx		8%		
University of Virginia	Total	162		Research	−3.1 ± 3.2
	# Yes fx	30			
	% fx	19%			
Residential centers^b^	Total	51		Research	−4.1 ± 2.3
	# Yes fx	7			
	% fx	14%			
All sites combined	Total	507	112		
	# Yes fx	149	19		
	% fx	29%	17%		

a Distal femur region 1 BMD *Z*-score; mean ± SD.

b Residential centers were the Hattie Larlham Center for Children with Disabilities, Mantua, OH, and the Children's Care Hospital and School, Sioux Falls, SD.

All DXA technologists conducting distal femur DXA scans received direct one-on-one training in the technique either at A. I. DuPont Hospital for Children, Wilmington, DE, or at their own center from the lead technologist at A. I. duPont (HHK). Following the initial training session, all technologists submitted a sample of their first 10 scans to A. I. duPont for quality-control evaluation. All centers use Hologic DXA scanners (Bedford, MA, USA). The scanners were older-generation pencil-beam models (QDR 1000 and 2000) in the early work from 1996 to 2001 at the two centers that developed the technique (University of North Carolina and A. I. duPont); subsequently, all eight centers have used fan-beam models (Delphi/Discovery, Bedford, MA, USA).

Indications for obtaining the DXA scan varied among centers. At some sites, scans were obtained most commonly because of clinical concerns over skeletal fragility; at other sites, the scans were obtained as part of broader clinical research projects focused on growth of children with disabilities. Consistent with this, bone density generally was lower and fracture prevalence higher at the centers where clinical concerns rather than clinical research prompted the scan (see Table 1). Some of the data obtained for clinical research has been published previously.(14,19) Informed consent under local Institutional Review Board (IRB) approval was obtained for scans done as part of clinical research projects. For this project, all sites obtained local IRB approval for centralized submission and review of their data without patient identifying information.

History of prior fracture was obtained at the time of the DXA scan from the subject and care provider(s) and was not consistently confirmed by review of medical records or radiographs. Information on date, anatomic location, mechanism of injury, treatment, and outcome of any reported fracture(s) was inconsistently obtained by history and/or the medical records and was of variable reliability when such data were collected. Therefore, the only fracture data reported herein are simple categorization of each subject as yes or no prior fracture.

### Distal femur DXA scan technique

The technique has been described previously.(12,13) Briefly, subjects are placed in the lateral position with the top limb flexed at the hip and knee so that it does not overlie the lower limb, which lies directly on the table and will be scanned. These children typically have hip and knee flexion contractures, so with the top limb supported on foam, this is usually a stable, comfortable position. The distal femur is scanned as a “forearm” and takes less than a minute with a fan-beam scanner. Motion while obtaining any DXA scan sometimes can be problematic in children who are uncooperative because of their young age or cognitive impairment or a motor disorder characterized by exaggerated startle reflexes or involuntary movements. Quiet surroundings and involving the child's care provider to sooth and hold the child can be helpful. Sedation is very rarely used at one of the eight centers to obtain a DXA scan in these children and never used at the other centers.

DXA scans of the distal femur were divided into three separate subregions for analysis. Region 1 is just proximal to the growth plate and consists almost exclusively of metaphyseal cancellous bone. Region 2 is immediately proximal to region 1 and covers the transition from the metaphysis to diaphysis. Region 3 is immediately proximal to region 2 and consists primarily of diaphyseal cortical bone. Each of these three subregions and the total L1–4 lumbar spine region were independently analyzed. For subjects who were followed longitudinally with serial DXA scans, only the first acceptable-quality distal femur DXA scan was used in the analyses. The right and left sides were averaged whenever acceptable-quality scans were obtained bilaterally.

Precision was assessed at two centers with a combined total of 30 subjects who underwent duplicate distal femur DXA scans of one lower limb on the same day. These subjects ranged in age from 5 to 17 years, and all had physical disabilities that impaired ambulation. The precision error expressed as a percent coefficient of variation (CV) as recommended by the International Society for Clinical Densitometry was 2.6% in region 1, 2.0% in region 2, and 2.1% in region 3 (%CV calculation tool available at http://www.iscd.org).

### BMD *Z*-scores

There are two sets of normal pediatric reference data for DXA measures of BMD in the distal femur. The initial reference series consisted of 256 subjects measured with Hologic pencil-beam scanners(13); the recent series includes over 800 subjects and used Hologic fan-beam scanners.(18) Areal BMD (aBMD) in each subregion of the distal femur and the lumbar spine was converted to an age- and gender-adjusted *Z*-score using one of these series of reference data. Owing to differences between fan- and pencil-beam measures of BMD,(18) the reference data used to calculate the *Z*-score were selected based on the type of scanner that had been used. Ninety-seven subjects had been scanned with pencil-beam models and 522 with fan-beam models. Reference data for nonblacks were used in the calculation of BMD *Z*-scores so that the BMD for all subjects would be scaled to the same age- and gender-specific BMD values.

### Statistical analysis

With aging, there is a greater risk for having sustained a fracture simply on the basis of longer risk exposure; 34% of the subjects 11 years of age or older had previously fractured as compared with 18% of those younger than 11 years of age (equality of proportions test, *p* < .0001). Further, BMD *Z*-scores decline with age, confirming previous reports that during growth these children fall farther from the norm.(5,20) For example, mean BMD *Z*-score was −3.3 ± 3.0 (region 2, ± SD) in subjects younger than 11 years of age compared with −5.1 ± 3.7 in subjects 11 years of age or older (two-sample *t* test, *p* < .0001). As a result of these two factors, age will indirectly link fracture prevalence and BMD *Z*-scores. To compensate for this, a survivorship analysis was used to test the likelihood that having sustained a previous fracture was correlated with BMD *Z*-scores (accelerated failure time model, Weibull distribution for failure times). A survivorship analysis adjusts for time at risk for fracture (i.e., the subject's age), thus allowing a more direct analysis of the effect of BMD *Z*-score on fracture risk.

## Results

Most of these children with impaired or no ability to ambulate had very low BMD *Z*-scores that were lower in the distal femur than in the lumbar spine (Table 2). In the subset of subjects with BMD measured at both sites, the median difference between a subject's lumbar spine BMD *Z*-score and lowest distal femur BMD *Z*-score was 2.0; in 34% of subjects, the lumbar spine BMD *Z*-score was at least 3.0 greater and in 76% at least 1.0 greater. One or more previous fractures had occurred in 149 of the 507 CP subjects (29%) and 19 of the 112 MD subjects (17%).

**Table 2:** BMD *Z*-scores^a^

	Distal femur			
		
	Region 1	Region 2	Region 3	Lumbar spine
All subjects	−4.0 ± 3.1	−4.3 ± 3.6	−3.2 ± 2.4	−2.3 ± 1.7
CP subset	−4.0 ± 3.1	−4.3 ± 3.3	−3.3 ± 2.5	−2.5 ± 1.7
MD subset	−3.8 ± 3.1	−4.3 ± 4.6	−2.6 ± 2.1	−1.7 ± 1.1

a Mean ± SD.

**Fig. 1:**
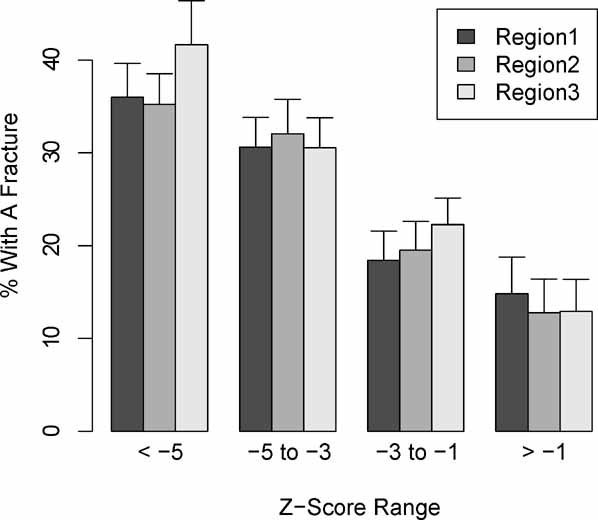
Fracture prevalence as a function of distal femur BMD *Z*-score.

The data are divided into five equal-sized groups for more detailed presentation in Table 3 and shown graphically in Fig. 1, categorized simply on round-number *Z*-scores. Note that Table 3 and Fig. 1 present the “raw” data, which include an indirect link between BMD *Z*-score and fracture based on age. Each subject's BMD *Z*-score in the three subregions of the distal femur were closely correlated but were only weakly correlated with the subject's lumbar spine BMD *Z*-score (Table 4).

**Table 3:** Prevalence of Fracture Versus BMD *Z*-Score

	Distal femur							
			
	Region 1		Region 2		Region 3		Lumbar spine	
				
Pentile group	*Z*-score	% fractured	*Z*-score	% fractured	*Z*-score	% fractured	*Z*-score	% fractured
1	> −1.8	13%	> −1.7	14%	> −1.4	11%	> −1.0	17%
(Highest *Z*-scores)		16 of 124		17 of 125		14 of 124		8 of 47
2	−1.8 to −3.2	23%	−1.7 to −3.0	21%	−1.4 to −2.5	24%	−1.0 to −1.9	26%
		29 of 124		26 of 124		30 of 123		12 of 46
3	−3.2 to −4.3	33%	−3.0 to −4.5	32%	−2.5 to −3.5	22%	−1.9 to −2.6	20%
		40 of 123		39 of 123		27 of 123		9 of 45
4	−4.3 to −5.8	37%	−4.5 to −6.5	31%	−3.5 to −4.8	37%	−2.6 to −3.5	39%
		46 of 124		38 of 124		45 of 123		18 of 46
5	< −5.8	30%	< −6.5	39%	< −4.8	41%	< −3.5	44%
(Lowest *Z*-scores)		36 of 122		48 of 123		50 of 122		20 of 45

**Table 4:** Correlation Between Distal Femur Subregions and Lumbar Spine *Z*-Scores^a^

	Distal femur			
		
	Region 1	Region 2	Region 3	Lumbar spine
Distal femur				
Region 1	1.00			
Region 2	0.72	1.00		
Region 3	0.72	0.81	1.00	
Lumbar spine	0.37	0.46	0.57	1.00

a Pearson's correlation coefficients.

The survivorship analyses assessed the more direct relationship between BMD *Z*-scores and fracture risk with the results expressed as a risk ratio, or the increased risk of fracture with each standard deviation decrease in BMD (a 1.0 decrease in BMD *Z*-score). The risk ratio for each region of the distal femur is given in Table 5, along with a range reflecting the 95% confidence interval. Risk of having sustained a fracture increased roughly 6% (region 2) to 15% (region 3) with each 1.0 drop in distal femur BMD *Z*-score. The differences between the three subregions were not statistically significant. The relationship between distal femur BMD *Z*-scores and fracture was consistent in both CP and MD subjects and across all enrollment centers; diagnosis (CP versus MD) and site of enrollment were not significant variables in the model. Unfortunately, the survivorship analysis could not be applied successfully to the lumbar spine data; the model failed to converge owing to an inadequate sample size.

**Table 5:** Risk Ratios for Fracture Risk Based on BMD *Z*-Scores^a^

	Risk ratio	95% Confidence interval	*p* Value
Distal femur			
Region 1	1.086	1.041–1.134	.0001
Region 2	1.063	1.024–1.102	.0006
Region 3	1.152	1.091–1.216	<.0001

a The increase in fracture risk for each SD deviation decrease in BMD (a 1.0 decrease in BMD *Z*-score).

## Discussion

Quadriplegic CP is the most prevalent pediatric condition with severe osteopenia. The prevalence of CP is 2 to 3 per 1000 live births, and 20% are involved to the extent that they are unable to ambulate.(21) Other pediatric disorders are also associated with severe motor impairment, including the muscular dystrophies, myelodysplasia (spina bifida), spinal cord injuries, cerebellar (Friedrich) ataxia, spinal muscular atrophy, Rett syndrome, and severe traumatic brain injury. Despite the wide variation in pathophysiologies, these conditions have in common skeletal fragility.

Undoubtedly, diminished ambulation is a major factor, but it is important to recognize that the etiology of skeletal fragility in these children is complex, resulting from the interplay of potentially multiple factors. For examples, BMD in the proximal femur but not the lumbar spine is severely diminished in boys with Duchenne MD early in the course of the disease before ambulation is significantly affected.(7) In persons with acute spinal cord injury, BMD is truly lost, but in children with CP, generally BMD increases over time despite declining BMD *Z*-scores(20); skeletal fragility in CP is part of a more complex growth disorder.(22) Nutritional factors and medications such as steroids and anticonvulsants can contribute to poor bone health in children with these conditions. Short-term immobilization for surgeries or fractures, diminished sunlight exposure, feeding difficulties, and altered pubertal progression also may be important factors in children with assorted physical disabilities.

Not only is the etiology of skeletal fragility complex in these children, so too is the assessment. Joint contractures, hip dysplasia, and metallic implants usually prevent reliable measures of BMD in the proximal femur; less commonly, scoliosis and spinal fusion instrumentation prevent DXA measures in the lumbar spine. It is critical to note in children with physical impairments that measures of BMD in the lumbar spine may not accurately reflect BMD in the femur. In this study, the correlation between BMD *Z*-scores in the lumbar spine and region 1 of the distal femur was only 0.37 compared with a correlation of 0.61 reported in normal children.(18) Other reports confirm the often large differences between BMD *Z*-scores in the femur and spine of children with low BMD.(7,14,23) In clinical practice with these children, one should not be falsely reassured by a lumbar spine BMD *Z*-score that is only mildly to moderately low; BMD *Z*-score in the femur is likely to be at least 1.0 lower, and in this series, one-third were at least 3.0 lower.

Technical difficulties are generally apparent when attempting to obtain an assessment of BMD. However, the more subtle issue routinely overlooked is whether the BMD assessment in a child with a particular condition is at all relevant to the clinical problem of fractures in that specific population. In children with physical impairments, the femur is the most common site of fracture,(7,9,10) and very rarely do they sustain spinal compression fractures. This, coupled with the weak correlation between BMD *Z*-scores in the femur and spine, likely explains the finding in a previous longitudinal study that BMD *Z*-scores in the lumbar spine did not predict fracture risk in a small series of 43 children and adolescents with CP and little or no ability to ambulate.(11) In this much larger study, the simple cross-sectional data (see Table 3) suggest that lumbar spine BMD *Z*-scores likely do correlate with fracture risk. However, the sophisticated survivorship analysis necessary to account for age could not be applied successfully to the lumbar spine data owing to an inadequate sample size. Thus the relationship between lumbar spine DXA measures and fractures in this population is currently best characterized as “unproven.”

Another complexity in the assessment of pediatric bone “density” relates to the fact that DXA provides measures of aBMD (g/cm^2^) rather than measures of true volumetric density (g/cm^3^). As a result, differences in bone size can significantly affect the measured aBMD independent of any differences in true volumetric density. This difference between aBMD and volumetric measures has resulted in the widespread practice of “correcting” or “adjusting” for size of pediatric subjects when interpreting DXA aBMD measurements. Typically, such adjustments are based on height of the child, but in nonambulatory children it is difficult to obtain an accurate measure of height owing to contractures, scoliosis, and the inability to stand erect. As a result, height measures were not consistently available for subjects in this series. However, it was found that simple age- and gender-normalized *Z*-scores for BMD in the distal femur, without consideration of subject size, correlated strongly with fracture. The issue of whether or not some sort of adjustment for size of the subject would strengthen the relationship between distal femur DXA measures and fracture warrants further investigation.

One of the limitations of this study was that the primary outcome (history of fracture) sometimes was based on self-report and not consistently confirmed by review of medical records or X-rays. Potentially, a past event may be erroneously recalled as a fracture when it was not, thus overreporting the outcome with false-positive results.(24) It is expected that the likelihood of this error would be independent of BMD *Z*-scores and thus not significantly affect the observed relationship between BMD *Z*-scores and fracture.

Recall self-report of fractures also may result in false-negative results with underreporting of fractures.(24) A fracture may have been recognized when it occurred but was simply forgotten when fracture history was later obtained. Further, several factors make it possible for fragility fractures in these children sometimes to go undiagnosed: (1) Such fractures may occur without significant or recognized trauma, (2) the child may be unable to effectively communicate, and (3) in osteopenic bone, a fracture that is minimally displaced or angulated can be difficult to identify on radiographs or clinical examination. For these reasons, it has been recommended that a bone scan be obtained in the evaluation of profoundly involved children who appear to be in pain of uncertain etiology, which is not a rare clinical dilemma in this population.(25) In that report, a bone scan identified a fragility fracture in 10 of 45 such children. These factors contributing to the underreporting of fractures are weighted toward those children with the lowest BMD *Z*-scores. Therefore, this bias would tend to diminish the observed relationship between distal femur BMD *Z*-scores and fracture.

This study in children with disabilities carries with it the same significant limitations as many of the early similar studies on osteoporotic fractures in postmenopausal women, and those studies have been critically reviewed.(26) One finding of that review was that the magnitude of the relationship varied more among cross-sectional studies than among prospective, longitudinal studies. The potential causes for this were bias in subject selection and/or postfracture bone loss and led the authors to recommend placing greater emphasis on prospective studies. Another recommendation from this review was to minimize subject selection bias by ensuring that nonfracture subjects indeed come from the same pool as fracture subjects. Fracture history was not a potential selection bias with roughly half of our subjects for whom BMD measures were obtained as part of broader clinical research projects focused on growth and nutrition in children with moderate to severe CP. Indeed, prospective studies of fractures and BMD in children with disabilities are warranted.

The technique of using DXA to assess BMD in the distal femur of children with severe motor impairment was developed to accomplish both technical feasibility and clinical relevance to fractures. This multicenter cross-sectional study supports our hypothesis that this technique provides measures that are clinically relevant in this population, with risk ratios of 1.06 to 1.15 for the different subregions of the distal femur. These findings, the technical feasibility of obtaining a reliable assessment of BMD in the distal femur, and the recent publication of more robust normal reference data(18) establish distal femur DXA as the clear technique of choice for assessment of BMD in children and adolescents with significantly impaired mobility. However, being the technique of choice is due far more to the lack of any more feasible, more available, or better validated alternatives than on the state of development of the distal femur technique. Prospective longitudinal studies are necessary to truly establish the predictive value of these measures, and the potential impact of bone and body size issues on the relationship to fracture risk warrants study. This study was limited to subjects up to 18 years of age, in keeping with the upper limit of the available normal reference data. Application of the technique to adults with disabilities and broadening of the normal reference age range are additional important future steps.
